# CCESC: A Crisscross-Enhanced Escape Algorithm for Global and Reservoir Production Optimization

**DOI:** 10.3390/biomimetics10080529

**Published:** 2025-08-12

**Authors:** Youdao Zhao, Xiangdong Li

**Affiliations:** Faculty of Land Resource Engineering, Kunming University of Science and Technology, Kunming 650500, China; kust_zyd@163.com

**Keywords:** escape algorithm, crisscross, production optimization, metaheuristic, bio-inspired optimization

## Abstract

Global optimization problems, ubiquitous scientific research, and engineering applications necessitate sophisticated algorithms adept at navigating intricate, high-dimensional search landscapes. The Escape (ESC) algorithm, inspired by the complex dynamics of crowd evacuation behavior—where individuals exhibit calm, herding, or panic responses—offers a compelling nature-inspired paradigm for addressing these challenges. While ESC demonstrates a strong intrinsic balance between exploration and exploitation, opportunities exist to enhance its inter-agent communication and search trajectory diversification. This paper introduces an advanced bio-inspired algorithm, termed Crisscross Escape Algorithm (CCESC), which strategically incorporates a Crisscross (CC) information exchange mechanism. This CC strategy, by promoting multi-directional interaction and information sharing among individuals irrespective of their behavioral group (calm, herding, panic), fosters a richer exploration of the solution space, helps to circumvent local optima, and accelerates convergence towards superior solutions. The CCESC’s performance is extensively validated on the demanding CEC2017 benchmark suites, alongside several standard engineering design problems, and compared against a comprehensive set of prominent metaheuristic algorithms. Experimental results consistently reveal CCESC’s superior or highly competitive performance across a wide array of benchmark functions. Furthermore, CCESC is effectively applied to a complex reservoir production optimization problem, demonstrating its capacity to achieve significantly improved Net Present Value (NPV) over other established methods. This successful application underscores CCESC’s robustness and efficacy as a powerful optimization tool for tackling multifaceted real-world problems, particularly in reservoir production optimization within complex sedimentary environments.

## 1. Introduction

Optimization problems are fundamental to progress in a wide range of scientific disciplines and industrial sectors [1,2,3]. They address the ubiquitous challenge of identifying the most effective or efficient solution from a vast set of possibilities. The core objective is typically to enhance performance, minimize costs, or maximize a desired outcome [4]. Such problems are pervasive, with applications ranging from designing resilient engineering structures [5] and fine-tuning complex manufacturing processes to formulating profitable financial strategies [6] and optimizing resource allocation in environmental management [7,8]. However, translating real-world problems into tractable mathematical models often presents significant complexities. These challenges typically include high-dimensional search spaces, non-linear relationships, numerous local optima (multimodality), and intricate, often conflicting, constraints [9]. The complexity can be further compounded by dynamic or uncertain operating environments.

Historically, various traditional mathematical optimization methods have been developed to address these challenges [10]. Prominent among these are gradient-based algorithms, such as the steepest descent [11] and conjugate gradient methods [12], which leverage derivative information to iteratively approach an optimum. While highly effective for convex and continuously differentiable problems, their performance degrades significantly when encountering non-convex landscapes, discontinuities, or numerous local optima, where they are prone to converging to suboptimal solutions [13]. Other classical approaches include linear programming, which excels in well-defined linear systems [14], and dynamic programming, which can optimally solve certain sequential decision-making problems [15]. However, a critical limitation of many traditional techniques is their reliance on specific problem characteristics, such as differentiability, linearity, or convexity. Furthermore, they often struggle with high-dimensionality [16]. As the number of decision variables increases—a common feature of contemporary optimization tasks—these methods can suffer from the “curse of dimensionality” [17]. This phenomenon leads to an exponential increase in computational cost, which severely diminishes their ability to explore the vast search space and renders them impractical for many complex, large-scale scenarios [18].

In response to these limitations, researchers have increasingly turned to biomimetics: the interdisciplinary field of science and engineering that learns from and mimics strategies found in nature to solve complex human problems. Within the computational domain, a powerful application of this principle is the development of metaheuristic algorithms, which have emerged as versatile alternatives for solving complex optimization problems [19]. Unlike traditional methods, metaheuristics are generally not constrained by a problem’s mathematical properties, such as convexity or differentiability [20]. Instead, they often draw inspiration from natural phenomena—such as evolutionary processes, swarm behaviors, or physical annealing—to implement stochastic search strategies. This inherent flexibility and their derivative-free nature enable them to effectively navigate rugged, multimodal search landscapes [21]. Consequently, they are particularly well-suited for high-dimensional, non-linear, and black-box optimization problems, where the analytical form of the objective function is either unknown or too complex to be used [22].

Metaheuristic algorithms are primarily categorized by their source of inspiration, with the most prominent classes being evolutionary, physics-based, and bio-behavioral algorithms [21]. Despite their diverse origins, these methods typically share a common operational framework: they initialize a population of candidate solutions and iteratively refine this population using specialized operators. These operators are designed to balance exploration of the search space with exploitation of promising regions, ultimately returning an optimal or near-optimal solution when a termination criterion is met. Evolutionary algorithms are inspired by Darwinian principles of natural selection and genetic inheritance [23]. Representative examples include the Genetic Algorithm (GA) [21] and Differential Evolution (DE) [24]. In contrast, physics-based algorithms draw analogies from physical laws. Notable examples include Simulated Annealing (SA) [25], which mimics the metallurgical annealing process, and the Gravitational Search Algorithm (GSA) [26]. Finally, bio-behavioral algorithms emulate the collective or individual behaviors of organisms. This category is dominated by swarm intelligence (SI) algorithms, which model decentralized problem-solving. Well-known SI methods include Particle Swarm Optimization (PSO) [27], Ant Colony Optimization (ACO) [28], and the Artificial Bee Colony (ABC) algorithm [29].

Despite the proliferation and success of diverse metaheuristic algorithms, the development of novel and more efficient optimization techniques remains an active area of research. This ongoing pursuit is fundamentally motivated by the “No Free Lunch” (NFL) theorem [30]. The NFL theorem posits that no single algorithm can universally outperform all others across the full spectrum of optimization problems [31]. In other words, an algorithm that excels at solving one class of problems (e.g., continuous, unimodal functions) may perform poorly on another (e.g., discrete, highly multimodal functions), and vice versa. This theorem highlights an inherent trade-off in algorithm design: the strategies that confer an advantage for certain problem structures inevitably result in disadvantages on others. Consequently, the NFL theorem underscores the necessity of developing new algorithms or enhancing existing ones. Such efforts are crucial for targeting the specific characteristics of particular problem domains or for addressing the identified weaknesses of current methods to improve superior practical performance [26].

Reservoir production optimization is a prominent and highly complex application domain where the limitations of traditional methods are apparent and metaheuristics have shown significant potential [32]. In petroleum engineering, the strategic management of oil and gas reservoirs is paramount to maximizing hydrocarbon recovery, ensuring economic viability, and minimizing environmental impact [33]. This optimization process involves complex decisions regarding well placement, drilling trajectories, injection strategies (e.g., water or gas flooding rates and locations), and individual well production rates over extended periods [34]. The primary objectives typically include maximizing the project’s Net Present Value (NPV), enhancing the Ultimate Oil Recovery (UOR), or maintaining specific production plateaus. Achieving these objectives, however, is exceptionally challenging. Reservoir systems are inherently characterized by significant geological heterogeneity, complex multiphase fluid dynamics, considerable subsurface uncertainty, and numerous operational constraints [35]. These factors result in highly non-linear, high-dimensional, and computationally expensive simulation models. Consequently, exhaustive search and traditional gradient-based approaches are often rendered impractical or ineffective for this class of problems.

Metaheuristic algorithms offer distinct advantages in navigating such complex problems. Their ability to perform a global search without relying on gradient information, coupled with their capacity to handle non-linearities and constraints (often through penalty functions or specialized mechanisms), makes them robust in the face of noisy or uncertain objective functions. These features render metaheuristics particularly well-suited for reservoir production optimization [36]. By effectively exploring the vast decision space, these algorithms can identify superior and often non-intuitive operational strategies. Ultimately, the development of robust and efficient optimization schemes for reservoir production yields substantial economic and operational benefits [37].

Scholarly efforts in reservoir production optimization have largely focused on two complementary avenues: enhancing computational efficiency and improving the core optimization algorithms. One stream of research aims to mitigate the high computational cost of full-physics simulations. An et al. [38] demonstrated this by integrating reservoir engineering principles into Particle Swarm Optimization (PSO), finding that using engineering knowledge to constrain the search space most effectively accelerated convergence, reducing iterations by over 24%. Others have bypassed full simulators entirely by using fast proxy models. For example, Gu et al. [39] employed an Extreme Gradient Boosting model to predict water cut, while Chen et al. [40] developed the Global and Local Surrogate-Model-Assisted Differential Evolution (GLSADE) algorithm, which iteratively refines a surrogate to focus the search. Both studies successfully coupled these surrogates with a DE optimizer to achieve improved outcomes with greater efficiency. A parallel research thrust focuses on enhancing the internal mechanisms of metaheuristic algorithms. Luo et al. [41] addressed premature convergence in the Water Flow Optimizer (WFO) by integrating a cross-search strategy, creating CCWFO, which yielded a higher Net Present NPV on a three-channel reservoir model. Similarly, Gao et al. [42] developed MGFOA by augmenting the Fruit Fly Optimization Algorithm (FOA) with multi-swarm and greedy selection mechanisms, leading to significantly better performance in oil and gas production optimization. Highlighting this trend, Li et al. [43] enhanced the bio-inspired Moss Growth Optimization (MGO) by incorporating a Crisscross (CC) strategy and dynamic grouping. Their resulting CCMGO algorithm not only excelled on CEC2017 benchmarks but also achieved a superior NPV in a reservoir application, explicitly demonstrating the value of improving an algorithm’s internal information exchange and adaptive balancing. While extensive research has focused on surrogate model development or heuristic integration, often defaulting to established optimizers like DE and PSO, the aforementioned studies reveal a critical insight: significant performance gains are consistently achieved by enhancing the core search behavior of the optimizer itself. The repeated success of hybridization, particularly with strategies like Crisscross search, underscores the pivotal role of the optimization engine’s architecture. This motivates the present work, which focuses not on modifying the simulation framework, but on advancing the state of the art by improving a novel, bio-inspired algorithm—the ESC algorithm—through a similar, powerful hybridization strategy.

The ESC algorithm, introduced by Chen et al. in 2025 [44], is a metaheuristic inspired by the dynamic behaviors observed during emergency crowd evacuations. Drawing from real-world crisis scenarios where individuals exhibit diverse responses, ESC translates these human behavioral dynamics into a computational optimization framework. At its core, particularly during the exploration phase, ESC partitions its solution population into three principal behavioral archetypes: the calm crowd, which promotes methodical search and provides guidance; the herding crowd, which facilitates convergence by following perceived group movement toward promising regions; and the panic crowd, which introduces erratic movements to enhance search diversity and escape local optima.

From an evolutionary computing perspective, the motivation for enhancing ESC stems from its unique, albeit flawed, approach to population diversity and information sharing. Many metaheuristics struggle to maintain a healthy balance between convergence (intensification) and diversity (diversification) throughout the search. ESC attempts to solve this by structurally segregating these functions into distinct subgroups (calm, herding, panic). This explicit partitioning is a notable strength, as it provides a clear framework for managing different search behaviors. However, this same structure introduces a significant limitation: the information flow between these subgroups is highly constrained and largely unidirectional (e.g., herding follows calm/panic). This segregation can lead to “information silos,” where good individuals discovered by one subgroup is not effectively propagated throughout the entire population, thereby limiting the generation of high-quality, diverse offspring and increasing the risk of premature convergence within a specific behavioral mode. The primary motivation for this work, therefore, is to address this structural weakness. We hypothesize that by introducing a mechanism that facilitates comprehensive information exchange across these behavioral subgroups, we can significantly enhance the algorithm’s overall performance.

In this paper, we introduce an enhanced bio-inspired metaheuristic known as the Crisscross Escape Algorithm (CCESC). The proposed CCESC method innovatively integrates the core framework of the ESC algorithm with a Crisscross (CC) strategy. The CC strategy, characterized by its capacity to facilitate multi-directional information exchange, generates offspring through interactions among individuals from different dimensions or population subgroups. This mechanism directly addresses the challenge of limited information flow within the original ESC algorithm. By promoting wider communication and more diverse solution generation across the entire population, regardless of an individual’s assigned behavioral role (i.e., calm, herding, or panic), the CC strategy enhances the ESC’s ability to explore uncharted areas, prevent premature convergence, and expedite the discovery of high-quality solutions.

The main contributions of this paper are summarized as follows:

- A novel CCESC algorithm is proposed, which synergistically integrates the crowd evacuation dynamics of ESC with a CC strategy to enhance search efficiency, solution diversity, and overall optimization performance.
- CCESC’s performance is rigorously validated on the CEC2017 benchmarks against numerous established metaheuristics, with statistical significance confirmed by Wilcoxon signed-rank and Friedman tests.
- The efficacy of CCESC in solving real-world problems is demonstrated through its application to reservoir production optimization, where it achieves a superior NPV and showcases its practical robustness.

The remainder of this paper is organized as follows. Section 2 briefly reviews the original ESC algorithm. Section 3 details the proposed CCESC algorithm and the integration of the Crisscross strategy. Section 4 presents the experimental setup, benchmark results, and statistical analyses. Section 5 demonstrates the application of CCESC to reservoir production optimization. Finally, Section 6 concludes the paper with a summary of the findings and suggestions for future research.

## 2. The Original ESC

The Escape algorithm (ESC), proposed in 2025 by Chen et al. [44], is a metaheuristic algorithm inspired by the complex dynamics of crowd behavior during emergency evacuations. ESC models the distinct responses observed in such scenarios by primarily dividing its population into three behavioral groups during its exploration phase: the calm crowd, representing rational search and guidance; the herding crowd, facilitating convergence by following collective movement; and the panic crowd, introducing randomness for broad exploration and escaping local optima. As the search progresses, the algorithm conceptually transitions towards an exploitation focus, where the population converges towards optimal solutions, analogous to a crowd finding the safest exit points. By simulating these diverse behavioral strategies, the core mathematical model of the ESC algorithm is structured as follows:

**1. Panic index:** To simulate the decreasing chaos as an evacuation progresses, a “Panic Index” P(t) is calculated at each iteration t. This index starts high and decreases over time, modulating the intensity of exploratory movements, particularly those influenced by panic or random adjustments. The P(t) can be calculated as follows:

(1) $$P(t)=cos(\frac{\pi t}{6T})$$

where t is current iteration, T is the maximum number of iterations.

**2. Calm crowd behavior:** Simulates rational individuals moving towards a collective decision point ($C_{j}$, the center of the calm group) while making minor adjustments ($v_{c,j}$). This promotes methodical search and guidance. A binary variable m1 introduces stochasticity, simulating potential “congestion” where some dimensions might not update. The movement is scaled by the P(t), and this behavior can be modeled as follows:

(2) $$x_{i,j}^{new}=x_{i,j}+m_{1}\times (w_{1}\times (C_{j}-x_{i,j})+v_{c,j})\times P(t)$$

where $C_{j}$ is the center (mean position) of the calm group in dimension j, $x_{i,j}^{new}$ is the updated position, $x_{i,j}$ is the current position, $m_{1}$ is a binary variable (0 or 1 with equal probability, Bernoulli distribution), $w_{1}$ is the adaptive Levy weight for dimension j, and $v_{c,j}$ is mathematically modeled as shown below:

(3) $$v_{c,j}=R_{c,j}-x_{i,j}+\epsilon_{j}$$

where $R_{c,j}=r_{min,j}^{c}+r_{i,j}\times (r_{max,j}^{c}-r_{min,j}^{c})$ is a randomly generated position within the calm group’s bounds, $r_{max,j}^{c}$ and $r_{min,j}^{c}$ respectively represent the lower and upper bounds of the j^th^ dimension for calm group. $\epsilon_{j}=\frac{z_{j}}{50}(z_{j}∼N(0,1))$ represents a slight adjustment in the individual’s movement.

**3. Herding crowd behavior:** The herding group’s individuals follow both the calm group’s direction ($C_{j}$) and the potentially disruptive influence of a random panic individual ($x_{pj}$). This balances convergence towards promising areas with the possibility of being pulled towards exploratory directions. This behavior can be modeled as follows:

(4) $$x_{i,j}^{new}=x_{i,j}+m_{1}\times (w_{1}\times (C_{j}-x_{i,j})+m_{2}\times w_{2}\times (x_{pj}-x_{i,j})+v_{h,j}\times P(t))$$

where $x_{pj}$ represents the j^th^ dimension of a randomly selected individual from the panic group, $m_{1}$ and $m_{1}$ are binary variables (0 or 1 with equal probability following a Bernoulli distribution), $w_{1}$ and $w_{2}$ denote the adaptive Levy weight for j^th^ dimension, and $v_{h,j}$ is the adjustment vector component specific to herding group bounds as defined above.

**4. Panic crowd behavior:** Simulates erratic individuals influenced by both potential exits and random directions. This mechanism drives exploration and diversification, helping to escape local optima. This behavior can be modeled as follows:

(5) $$x_{i,j}^{new}=x_{i,j}+m_{1}\times (w_{1}\times (E_{j}-x_{i,j})+m_{2}\times w_{2}\times (x_{rand,j}-x_{i,j})+v_{p,j}\times P(t))$$

where $E_{j}$ is the j^th^ dimension of a randomly selected individual from the Elite Pool, $x_{rand,j}$ represents the j^th^ dimension of a randomly selected individual from the population, and $v_{p,j}$ is the adjustment vector component specific to panic group bounds as defined above.

**5. Exploitation Phase:** In the second half of the iterations, the focus shifts entirely to exploitation. All individuals are treated as ‘calm’ and refine their positions by moving towards both elite solutions and random solutions. This simulates convergence towards identified optimal exits while maintaining some diversity to avoid stagnation. This phase can be modeled as follows:

(6) $$x_{i,j}^{new}=x_{i,j}+m_{1}\cdot w_{1}\cdot (E_{j}-x_{i,j})+m_{2}\cdot w_{2}\cdot (x_{rand,j}-x_{i,j})$$

**Figure 1:**
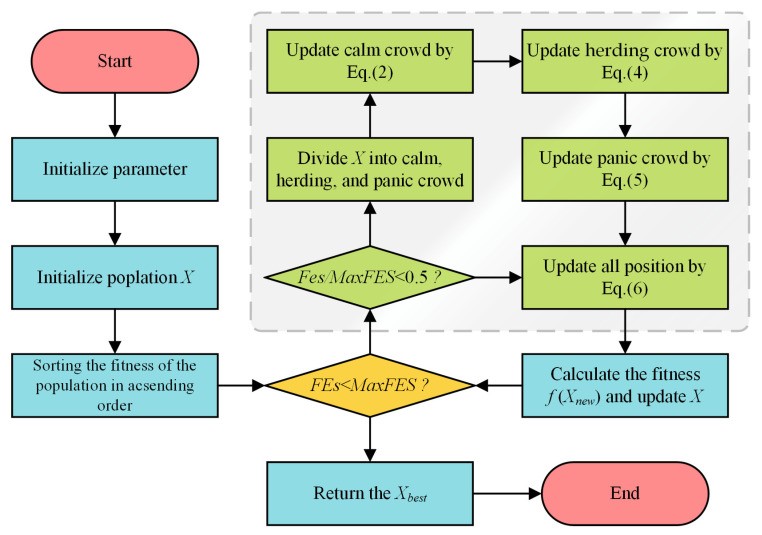
Flowchart of the ESC.

In summary, the ESC algorithm commences by initializing its parameters and randomly generating a population of N individuals within the defined search space. The fitness of each individual is then evaluated, the population is sorted, and the top existing individuals are stored in an Elite Pool E. The main iterative process then begins. Within each iteration t (from 1 to T), a Panic Index P(t) is computed (Equation (1)). If the algorithm is in the exploration phase (t/T ≤ 0.5), the population is sorted by fitness and divided into calm, herding (Conforming), and panic groups according to predefined proportions (c, h, p). Individuals in the calm group update their positions using Equation (2), herding group individuals use Equation (4), and panic group individuals use Equation (5). If the algorithm is in the exploitation phase (t/T > 0.5), all individuals update their positions using Equation (6). After these position updates, the fitness of each individual is re-evaluated, and a greedy selection is applied to retain the better solution between the old and new positions. Finally, the Elite Pool E is updated with the best solutions found in the current population. This iterative loop continues until the maximum number of iterations T is reached. The algorithm then returns the best solution found, typically the best individual residing in the Elite Pool. The flowchart of ESC is presented in Figure 1 of the original paper.

## 3. Proposed CCESC

### 3.1. Crisscross Strategy

To improve the ESC algorithm’s ability to avoid local optima and enhance its search capabilities, we incorporate the CC strategy, originally introduced in the Crisscross Optimization (CSO) algorithm [41]. The CC strategy facilitates superior information exchange within the population through two core mechanisms: Horizontal Crossover Search (HCS), which enables interaction between different individual solutions, and Vertical Crossover Search (VCS), which performs crossover across different dimensions of a single solution. Philosophically, this methodology is inspired by the “Doctrine of the Mean,” as it dynamically adjusts the search trajectory. The synergy between its unique crossover and selection methods enhances the algorithm’s global exploration and accelerates its convergence rate. To ensure a standard and robust implementation, the control parameters governing the CC strategy are adopted based on recommendations and common practices from the original CSO literature. The HCS and VCS mechanisms are detailed below.

**Horizontal Crossover Search:** The HCS operator enhances the algorithm’s exploratory performance by leveraging information from the entire population. The procedure involves randomly selecting pairs of particles and performing a crossover operation to generate new candidate solutions. The mathematical formulation for HCS is given by Equations (7) and (8).

(7) $${HCS}_{i}^{j}=r_{1}\times x_{i}^{j}+\left(1-r_{1}\right)\times x_{k}^{j}+c_{1}\times \left(x_{i}^{j}-x_{k}^{j}\right)$$

(8) $${HCS}_{k}^{j}=r_{2}\times x_{k}^{j}+\left(1-r_{2}\right)\times x_{i}^{j}+c_{2}\times \left(x_{k}^{j}-x_{i}^{j}\right)$$

where $x_{k}^{j}$ and $x_{i}^{j}$ are the j^th^ dimension of two distinct parent particles, the terms $r_{1}$ and $r_{2}$ are random numbers uniformly distributed in [0, 1], while $c_{1}$ and $c_{2}$ are random numbers uniformly distributed in [−1, 1], and ${HCS}_{i}^{j}$ and ${HCS}_{k}^{j}$ are the j^th^ dimension of new offspring generated by the HCS from the two particles.

**Vertical Crossover Search:** The VCS operator enhances the algorithm’s exploitation capabilities by leveraging information within a single individual. The procedure involves randomly selecting two distinct dimensions within each particle and performing a crossover between them. The mathematical formulation for VCS is as follows:

(9) $${VCS}_{i}^{j}=r_{3}\times x_{i}^{d1}+\left(1-r_{3}\right)\times x_{i}^{d2}$$

where $r_{3}$ is a random number distributed in [0, 1], $x_{i}^{d1}$ and $x_{i}^{d2}$ are the values of the two dimensions randomly picked by the i^th^ individual, ${VCS}_{i}^{j}$ denotes the newly generated value for a target dimension j. Similar to HCS, VCS employs a greedy selection strategy to determine whether the updated particle (with the new dimension value) replaces the original.

### 3.2. The Proposed CCESC

This section details the framework of the proposed CCESC. The algorithm begins by initializing its parameters and generating an initial population, following the standard procedure of the original ESC algorithm. Subsequently, the main iterative loop commences, where population updates are performed based on the ESC framework. This includes partitioning the population into calm, herding, and panic groups during the exploration phase and transitioning to a unified exploitation phase as the search progresses.

The key innovation of CCESC lies in the strategic integration of the CC strategy. By applying the CC strategy after the standard ESC-based behavioral updates, the algorithm facilitates multi-directional information exchange across the entire population, regardless of an individual’s assigned behavioral role (i.e., calm, herding, or panic). This mechanism enhances the ESC’s ability to explore uncharted areas, prevent premature convergence, and expedite the discovery of high-quality solutions. As will be verified by the experimental results in Section 4.2, this synergistic process leads to tangible improvements in overall optimization performance.

**Figure 2:**
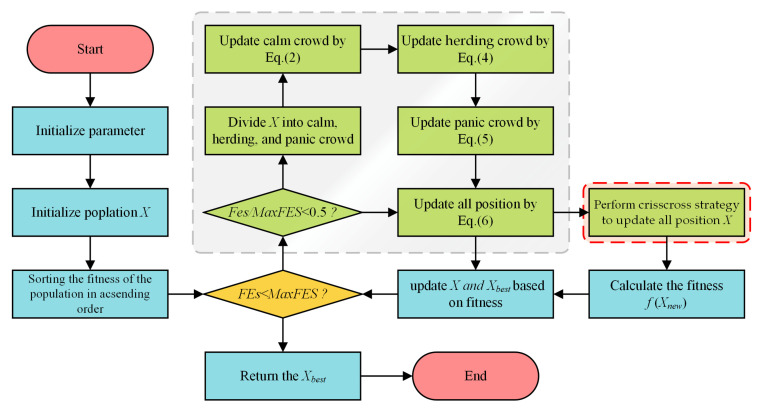
Flowchart of the CCESC.

Within each iteration, after the primary ESC-based behavioral updates are completed, the CC strategy is applied to the current population. By facilitating enhanced information exchange—both between individuals from different behavioral groups (Horizontal Crossover) and within individuals across different dimensions (Vertical Crossover)—the CC mechanism generates a new set of candidate solutions. This synergistic process, combining ESC’s behavioral mechanics with the CC strategy’s diversification and intensification capabilities, continues until a predefined termination criterion (e.g., the maximum number of function evaluations) is met. The overall workflow of CCESC is illustrated in Figure 2.

Algorithm 1 provides the pseudo-code for the CCESC.
**Algorithm 1** Pseudo-code of the CCESCSet parameters: MaxFEs, dim, population size N Initialize population X FEs = 0 **For** i=1:N    **Evaluate** the fitness value of $x_{i}$    **Find** the global min $x_{best}$ and fitness **End For** **Sort** population by fitness in ascending order **Store** the top individuals in the Elite Pool E **While**
(FEs<MaxFEs)       **IF** FEs/MaxFEs<0.5       **Generate** Panic Index by Equation (1)       **Divide** population into: Calm group (proportion c), Conforming group       (proportion h), and Panic group (proportion p)       **Update** Calm Group by Equation (2)       **Update** Herding crowd by Equation (4)       **Update** Panic Group by Equation (5)
       **ELSE**
       **Update** population by Equation (6)
       **End IF**
       FEs=FEs+N       **For** i=1:N                         /*CC*/
       **Perform** HCS to update $x_{i}$
       **Perform** VCS to update $x_{i}$
       **Update** $x_{best}$
       **End For**
       FEs=FEs+N
**End While**
**Return** $x_{best}$ **End**

The computational complexity of the CCESC algorithm is derived from four principal operations: population initialization, fitness function evaluation, position updates, and the CC strategy. The complexities associated with initialization and fitness evaluation are O (T × N), whereas position updates and the CC strategy exhibit a complexity of O (T × N × D). Consequently, the total computational burden is dominated by the higher-order term, yielding an overall complexity of O (T × N × D).

## 4. Experimental Results and Analysis

The performance of the proposed CCESC was evaluated using the 29 benchmark functions from the CEC2017 test suite. All experiments were conducted in MATLAB R2024a on a workstation with an Intel Xeon Gold 6258R CPU and 128 GB of RAM. For a fair and direct comparison, the experimental parameters were set to align with recent literature [41]: a population size (N) of 30, a problem dimension (D) of 30, and a maximum of 300,000 function evaluations (MaxFEs) as the termination criterion. The focus on 30 dimensions was intentionally chosen to provide a relevant benchmark for this study’s core application—a 40-dimension oil reservoir problem—thus ensuring the analysis remains clear and concise. To account for the stochastic nature of metaheuristics, 30 independent runs were performed for each function, with the mean and standard deviation (Std) of the final objective values recorded for analysis.

### 4.1. Benchmark Functions Overview

The benchmark problems employed in this study are drawn from the CEC2017 test suite and are categorized into four distinct types: unimodal, simple multimodal, hybrid, and composition functions. This suite, comprising 29 functions in total, is designed to provide a comprehensive evaluation of an algorithm’s performance across a diverse range of optimization landscapes. A detailed overview of these benchmark functions is provided in Table 1.

### 4.2. Performance Comparison with Other Algorithms

This section presents a comparative performance evaluation of the proposed CCESC against the original ESC and eight other well-established metaheuristics on the 29 benchmark functions from the CEC2017 suite. The selected peer algorithms include DE [24], GWO [45], MFO [46], SCA [47], PSO [27], the Parrot Optimizer (PO) [48], BA [49], and FA [50]. All algorithms were implemented according to their original publications, and their control parameters were configured based on common recommendations in the literature to ensure a fair comparison.

Table 2 provides a comprehensive comparison of these ten algorithms on the CEC2017 benchmark suite. For each algorithm and function, the table reports the average fitness (Mean) and standard deviation (Std Dev) obtained over 30 independent runs. To facilitate an overall performance assessment, the table also includes the final rank of each algorithm as determined by the Friedman test. Additionally, a pairwise statistical summary (+/=/−) is provided, indicating whether CCESC performed significantly better than (+), statistically similar to (=), or worse than (−) the original ESC on each function. This +/=/− comparison is based on the Wilcoxon signed-rank test conducted at a significance level of α = 0.05. All best values have been highlighted in bold.

The results demonstrate that CCESC achieves the best overall performance, attaining the lowest average Friedman rank of 2.1034. This places it ahead of ESC, which secured the second-best rank of 2.4828. The pairwise comparison reveals that CCESC significantly outperformed ESC on 11 functions, was statistically equivalent on 9, and was outperformed on the remaining 9. More broadly, the statistical tests confirm that CCESC achieves significant improvements (*p* < 0.05) over most competitors—including GWO, MFO, SCA, PO, BA, and FPA—on the vast majority of the 29 functions. It also secures compelling, statistically significant victories against other strong performers like DE and PSO on multiple problems. These findings confirm that the architectural modifications introduced in CCESC lead to tangible and verifiable enhancements. Furthermore, the standard deviation values for CCESC were consistently competitive, suggesting robust and stable performance.

**Figure 3:**
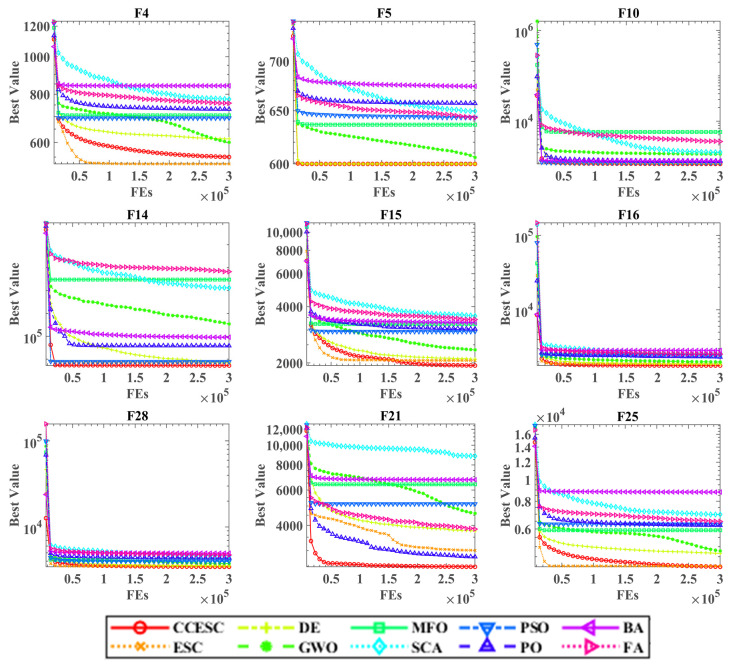
Convergence curves of the CCESC on benchmarks with other algorithms.

Figure 3 displays the convergence curves of all ten algorithms on nine representative benchmark functions (F4, F5, F10, F14, F15, F16, F21, F25, and F28), with CCESC highlighted by a red line. The horizontal axis represents function evaluations (FEs) up to 3 × 10^5^, and the vertical axis shows the best fitness achieved. The variation in initial fitness values across algorithms is an expected outcome of their random initialization processes and distinct internal strategies. The random seed was intentionally not fixed during experiments to ensure the observed performance is robust and generalizable, rather than being tied to a specific starting condition. A logarithmic scale is used for the *y*-axis on F10, F14, F16, and F28.

The convergence plots demonstrate CCESC’s superior performance. On most functions (e.g., F4, F5, F15, F21, and F25), CCESC converges faster and to significantly better solutions than both ESC and other competitors. This is evidenced by its rapid initial fitness decline, confirming effective early exploration, and a sustained lead throughout the search process. Even on the logarithmically scaled functions, CCESC either finds the best solution or remains highly competitive (e.g., F10, F14). In nearly all cases, CCESC’s curve lies below that of the original ESC, with the performance gap being particularly pronounced on F15 and F21. These results visually confirm that the integrated CC strategy significantly enhances both the convergence speed and solution quality of the algorithm.

In summary, the convergence analysis visually confirms that incorporating the CC strategy enhances the search performance of the original ESC algorithm. This enhancement enables CCESC to locate higher-quality solutions more efficiently and robustly across a diverse set of benchmark problems.

## 5. Application to Production Optimization

The goal of optimizing reservoir production is to identify the best control parameters for each well to maximize NPV. However, the extensive number of wells and production cycles results in a combinatorial explosion of potential solutions, leading to a high-dimensional, NP-hard optimization problem. Due to this complexity, evolutionary algorithms are particularly effective for addressing such issues. In this research, we employ the MRST reservoir simulator to implement CCESC on a reservoir model and evaluate its performance compared to several commonly used metaheuristic algorithms. For this study, non-linear constraints related to oilfield production are not considered, and the NPV, as specified in Equation (16), is used as the objective function.

(10) $$NPV\left(x,z\right)=\sum_{t=1}^{n}\;\Delta t\frac{Q_{o,t}\cdot r_{o}-Q_{w,t}\cdot r_{w}-Q_{i,t}\cdot r_{i}}{{\left(1+b\right)}^{p_{t}}}$$

where x is the solution to be optimized, z is the model state parameters, n is the total simulation time, $Q_{o,t}$, $Q_{w,t}$, $Q_{i,t}$ correspond to the oil production rate, water production rate, and water injection rate, respectively. $r_{o}$, $r_{w}$, and $r_{i}$ denote the oil revenue, and the cost of treating and injecting the water, respectively, b is the annual interest rate, $p_{t}$ is the cumulative time in years up to step t.

### 5.1. Reservoir Model Description

The reservoir model used in this study is a two-dimensional, heterogeneous synthetic system designed to represent a clastic depositional environment, such as a fluvial or deltaic channel system. The well configuration consists of four injection wells (INJ1–INJ4) and one central production well (PRO1), arranged in a modified five-spot pattern typical for such geological settings. The model domain is discretized into a 25 × 25 × 1 Cartesian grid, resulting in 625 active grid blocks. Each block has planar dimensions of 20 m × 20 m and a uniform thickness of 30 m. A constant porosity of 0.1 is assumed for all grid blocks, representing a moderately sorted sandstone reservoir.

**Figure 4:**
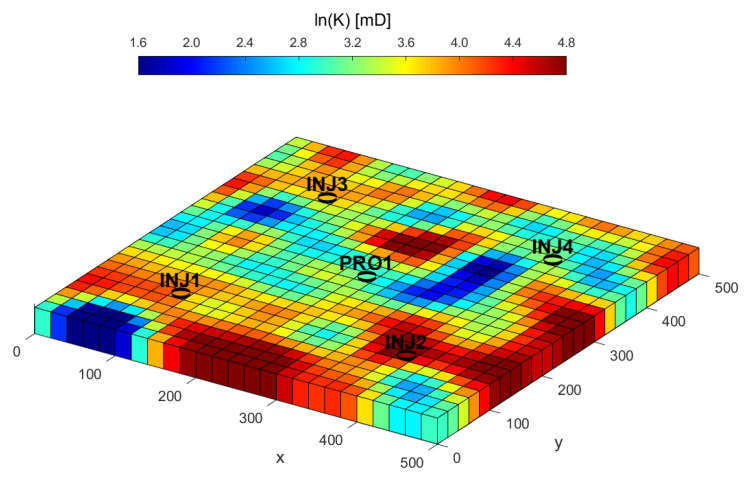
Distribution of log-permeability in the three-channel model.

The reservoir’s heterogeneity is principally defined by its permeability field, which was stochastically generated using the Karhunen–Loève (KL) expansion method. The natural logarithm of permeability, ln(K), was modeled with a mean of 3.5 and a standard deviation of 1.0. The resulting spatial distribution of log-permeability, depicted in Figure 4, reveals distinct high- and low-permeability channels that govern the fluid flow paths. For this study, a single-phase fluid (water) with a viscosity of 0.9 cP and a density of 1000 kg/m^3^ is considered.

The objective of this production optimization problem is to maximize the Net Present Value (NPV). The decision variables are the constant water injection rates for each of the four injection wells, which are optimized over a total simulation period of 2000 days. This period is divided into 10 discrete control steps, each with a duration of 200 days. The allowable injection rate for each well is constrained to the range of [0, 1000] m^3^/day. The single production well (PRO1) is operated at a constant bottom-hole pressure (BHP) of 200 barsa. Consequently, the optimization problem involves determining the optimal injection rate for each of the 4 wells across all 10 control steps, resulting in a 40-dimensional search space (4 wells × 10 steps).

The NPV serves as the fitness function and is calculated based on the following economic parameters: an oil price of 80.0 USD/STB, a water injection cost of 5.0 USD/STB, and a water treatment cost of 5.0 USD/STB. For simplicity, a discount rate of 0% per annum is assumed in this study.

### 5.2. Analysis and Discussion of Experimental Results

This section presents a detailed performance analysis of the proposed CCESC and six other metaheuristics—ESC, DE, GWO, MFO, SCA, and PSO—on the reservoir production optimization problem. To ensure a robust comparison for this computationally intensive application, each algorithm was executed 10 times independently. The optimization was performed for 100 iterations, and key statistical metrics, including the mean, standard deviation (Std Dev), best, and worst Net Present Value (NPV) achieved, are reported in Table 3.

The results summarized in Table 3 clearly demonstrate the superior performance of CCESC. It achieved the highest mean NPV of 9.457 × 10^8^ USD, underscoring its enhanced capability to consistently identify high-value production strategies for this complex reservoir model. Furthermore, CCESC exhibited a relatively low standard deviation of USD 1.532 × 10^7^, indicating greater stability and reliability compared to most of its peers, including DE (3.121 × 10^7^), GWO (2.784 × 10^7^), MFO (4.542 × 10^7^), SCA (3.487 × 10^7^), and PSO (3.939 × 10^7^). While the original ESC also showed competitive stability (Std Dev of 2.510 × 10^7^), its mean NPV was lower. These numerical results highlight CCESC’s dual strengths: it effectively explores the high-dimensional decision space of the reservoir model while efficiently exploiting promising regions to maximize economic returns with high consistency.

**Figure 5:**
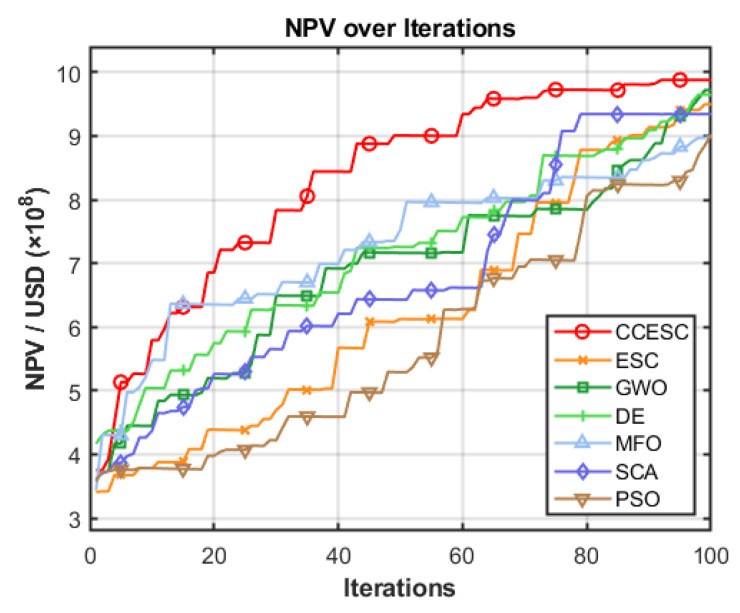
Convergence of NPV values for different algorithms over iterations.

Figure 5 illustrates the convergence history of the NPV for CCESC and the six other algorithms over 100 iterations. The horizontal axis represents the iteration number, while the vertical axis shows the corresponding best NPV achieved, scaled by USD 10^8^.

CCESC, represented by the red curve, exhibits the most rapid convergence and consistently attains the highest NPV. It demonstrates a steep initial improvement, surpassing an NPV of USD 9.0 × 10^8^ in fewer than 40 iterations, and continues to refine its solution toward the optimal value. In contrast, the original ESC shows good initial progress but quickly plateaus at a suboptimal level. While GWO and DE also display strong initial convergence, outperforming ESC in the early and middle stages, they ultimately yield lower final NPVs than CCESC. MFO and SCA converge more gradually, reaching moderate NPVs, whereas PSO exhibits the slowest convergence and achieves the lowest final NPV among all tested algorithms.

In summary, the reservoir production optimization case study highlights the practical advantages of the proposed CCESC. The algorithm not only achieves the highest average NPV but also converges faster and with greater stability than the original ESC and the other competing metaheuristics. These results confirm CCESC’s superior performance and its suitability for solving complex, real-world engineering optimization problems such as reservoir management.

## 6. Conclusions

This paper introduced the CCESC algorithm, a novel metaheuristic that enhances the original ESC algorithm by integrating a CC strategy. The ESC algorithm, inspired by crowd evacuation dynamics, balances exploration and exploitation by partitioning its population into calm, herding, and panic groups. The CC strategy augments this framework by promoting comprehensive information exchange across the entire population, thereby improving solution diversity and accelerating convergence, particularly on complex optimization landscapes.

The performance of CCESC was rigorously evaluated on the CEC2017 benchmark suite against the original ESC and eight other well-established metaheuristics. The experimental results demonstrated that CCESC achieved superior or highly competitive performance. Statistical analyses, including the Friedman and Wilcoxon signed-rank tests, confirmed that CCESC significantly outperformed the original ESC and many peer algorithms in terms of both solution accuracy and convergence speed. Furthermore, the practical efficacy of CCESC was validated on a challenging heterogeneous reservoir production optimization problem. In this real-world application, CCESC obtained a higher Net Present Value (NPV) and exhibited more robust convergence than the other tested algorithms, underscoring its potential for solving complex engineering tasks.

Future research will pursue several promising directions. First, we will investigate adaptive mechanisms for the control parameters of the CC strategy to further enhance performance. Second, extending CCESC to solve multi-objective optimization problems represents a key area for future development. Finally, we plan to apply CCESC to other complex, real-world challenges to further validate its versatility. These applications include optimizing robot parameters for impedance learning in human-guided interaction with unknown environments, Unmanned Aerial Vehicle (UAV) path planning, and hyperparameter tuning for deep learning models.

## Figures and Tables

**Table 1:** CEC2017 benchmark functions.

Function	Function Name	Class	Optimum
F1	Shifted and Rotated Bent Cigar Function	Unimodal	100
F2	Shifted and Rotated Zakharov Function	Unimodal	300
F3	Shifted and Rotated Rosenbrock’s Function	Multimodal	400
F4	Shifted and Rotated Rastrigin’s Function	Multimodal	500
F5	Shifted and Rotated Expanded Schaffer’s F6 Function	Multimodal	600
F6	Shifted and Rotated Lunacek Bi-Rastrigin Function	Multimodal	700
F7	Shifted and Rotated Non-Continuous Rastrigin’s Function	Multimodal	800
F8	Shifted and Rotated Lévy Function	Multimodal	900
F9	Shifted and Rotated Schwefel’s Function	Multimodal	1000
F10	Hybrid Function 1 (N = 3)	Hybrid	1100
F11	Hybrid Function 2 (N = 3)	Hybrid	1200
F12	Hybrid Function 3 (N = 3)	Hybrid	1300
F13	Hybrid Function 4 (N = 4)	Hybrid	1400
F14	Hybrid Function 5 (N = 4)	Hybrid	1500
F15	Hybrid Function 6 (N = 4)	Hybrid	1600
F16	Hybrid Function 6 (N = 5)	Hybrid	1700
F17	Hybrid Function 6 (N = 5)	Hybrid	1800
F18	Hybrid Function 6 (N = 5)	Hybrid	1900
F19	Hybrid Function 6 (N = 6)	Hybrid	2000
F20	Composition Function 1 (N = 3)	Composition	2100
F21	Composition Function 2 (N = 3)	Composition	2200
F22	Composition Function 3 (N = 4)	Composition	2300
F23	Composition Function 4 (N = 4)	Composition	2400
F24	Composition Function 5 (N = 5)	Composition	2500
F25	Composition Function 6 (N = 5)	Composition	2600
F26	Composition Function 7 (N = 6)	Composition	2700
F27	Composition Function 8 (N = 6)	Composition	2800
F28	Composition Function 9 (N = 3)	Composition	2900
F29	Composition Function 10 (N = 3)	Composition	3000

**Table 2:** Results of the CCESC and other algorithms on CEC2017.

	F1		F2		F3	
	**Avg**	**Std**	**Avg**	**Std**	**Avg**	**Std**
CCESC	3.5255 × 10^3^	3.8954 × 10^3^	1.0835 × 10^3^	5.3427 × 10^2^	4.7483 × 10^2^ =	2.9002 × 10^1^
ESC	3.3872 × 10^3^ =	3.9748 × 10^3^	6.3777 × 10^2^ −	4.5533 × 10^2^	4.6240 × 10^2^ +	3.0354 × 10^1^
DE	**2.7647 × 10^3^ =**	4.4090 × 10^3^	1.9190 × 10^4^ +	5.0403 × 10^3^	4.8943 × 10^2^ +	8.7689 × 10^0^
GWO	1.4755 × 10^9^ +	1.1091 × 10^9^	3.1543 × 10^4^ +	1.0232 × 10^4^	6.1372 × 10^2^ +	1.0357 × 10^2^
MFO	1.0161 × 10^10^ +	7.5321 × 10^9^	9.2600 × 10^4^ +	6.6262 × 10^4^	1.3525 × 10^3^ +	7.9515 × 10^2^
SCA	1.2752 × 10^10^ +	2.3869 × 10^9^	3.8462 × 10^4^ +	7.0213 × 10^3^	1.4624 × 10^3^ +	3.2765 × 10^2^
PSO	3.1524 × 10^3^ =	3.0479 × 10^3^	3.0000 × 10^2^ −	4.8342 × 10^−3^	**4.6206 × 10^2^ −**	2.5274 × 10^1^
PO	4.2669 × 10^7^ +	5.6668 × 10^7^	5.6686 × 10^3^ +	3.8437 × 10^3^	5.2065 × 10^2^ +	2.3061 × 10^1^
BA	5.5491 × 10^5^ +	2.4723 × 10^5^	**3.0010 × 10^2^ −**	7.6706 × 10^−2^	4.7381 × 10^2^ =	3.5388 × 10^1^
FA	1.4343 × 10^10^ +	1.4273 × 10^9^	6.1478 × 10^4^ +	1.0700 × 10^4^	1.3581 × 10^3^ +	1.2564 × 10^2^
	**F4**		**F5**		**F6**	
	**Avg**	**Std**	**Avg**	**Std**	**Avg**	**Std**
CCESC	5.5037 × 10^2^	7.8167 × 10^0^	**6.0000 × 10^2^**	6.5666 × 10^−6^	7.9386 × 10^2^	9.0650 × 10^0^
ESC	**5.2816 × 10^2^ −**	7.8871 × 10^0^	6.0010 × 10^2^ +	1.1095 × 10^−1^	**7.6211 × 10^2^ −**	9.3598 × 10^0^
DE	6.1306 × 10^2^ +	8.8681 × 10^0^	**6.0000 × 10^2^ =**	0.0000 × 10^0^	8.4353 × 10^2^ +	7.2832 × 10^0^
GWO	6.0056 × 10^2^ +	2.4028 × 10^1^	6.0595 × 10^2^ +	3.0534 × 10^0^	8.5675 × 10^2^ +	3.3805 × 10^1^
MFO	7.0553 × 10^2^ +	4.2114 × 10^1^	6.3645 × 10^2^ +	1.1182 × 10^1^	1.2015 × 10^3^ +	2.3191 × 10^2^
SCA	7.7670 × 10^2^ +	1.7574 × 10^1^	6.4961 × 10^2^ +	4.5914 × 10^0^	1.1169 × 10^3^ +	3.3466 × 10^1^
PSO	6.9392 × 10^2^ +	4.1274 × 10^1^	6.4342 × 10^2^ +	6.5115 × 10^0^	1.0271 × 10^3^ +	6.1418 × 10^1^
PO	7.3187 × 10^2^ +	4.6764 × 10^1^	6.5719 × 10^2^ +	7.5367 × 10^0^	1.1326 × 10^3^ +	7.4220 × 10^1^
BA	8.4090 × 10^2^ +	7.4849 × 10^1^	6.7434 × 10^2^ +	7.0809 × 10^0^	1.6100 × 10^3^ +	1.8629 × 10^2^
FA	7.5838 × 10^2^ +	1.1129 × 10^1^	6.4328 × 10^2^ +	3.3706 × 10^0^	1.3901 × 10^3^ +	3.4637 × 10^1^
	**F7**		**F8**		**F9**	
	**Avg**	**Std**	**Avg**	**Std**	**Avg**	**Std**
CCESC	8.5047 × 10^2^	6.8607 × 10^0^	9.0126 × 10^2^	1.2168 × 10^0^	3.5882 × 10^3^	2.4471 × 10^2^
ESC	**8.2653 × 10^2^ −**	6.7751 × 10^0^	9.2665 × 10^2^ +	2.1323 × 10^1^	**3.2137 × 10^3^ −**	7.9416 × 10^2^
DE	9.1027 × 10^2^ +	6.2547 × 10^0^	**9.0000 × 10^2^ −**	9.6743 × 10^−14^	5.8136 × 10^3^ +	2.8900 × 10^2^
GWO	8.8931 × 10^2^ +	2.1397 × 10^1^	1.7638 × 10^3^ +	6.3256 × 10^2^	4.0596 × 10^3^ +	6.3352 × 10^2^
MFO	1.0012 × 10^3^ +	4.9928 × 10^1^	7.6299 × 10^3^ +	2.2116 × 10^3^	5.5813 × 10^3^ +	9.2648 × 10^2^
SCA	1.0519 × 10^3^ +	1.7228 × 10^1^	5.2489 × 10^3^ +	1.0536 × 10^3^	8.1075 × 10^3^ +	3.3474 × 10^2^
PSO	9.4191 × 10^2^ +	2.4900 × 10^1^	4.0330 × 10^3^ +	5.9325 × 10^2^	4.8212 × 10^3^ +	5.3623 × 10^2^
PO	9.8016 × 10^2^ +	3.0531 × 10^1^	5.0865 × 10^3^ +	7.6591 × 10^2^	5.7686 × 10^3^ +	7.8686 × 10^2^
BA	1.0472 × 10^3^ +	5.6682 × 10^1^	1.2756 × 10^4^ +	4.4659 × 10^3^	5.4802 × 10^3^ +	6.2913 × 10^2^
FA	1.0552 × 10^3^ +	1.3119 × 10^1^	5.2547 × 10^3^ +	5.3521 × 10^2^	8.0185 × 10^3^ +	2.9787 × 10^2^
	**F10**		**F11**		**F12**	
	**Avg**	**Std**	**Avg**	**Std**	**Avg**	**Std**
CCESC	**1.1399 × 10^3^**	2.6975 × 10^1^	**3.0823 × 10^5^**	2.0745 × 10^5^	1.3200 × 10^4^	1.5007 × 10^4^
ESC	1.1684 × 10^3^ +	3.6141 × 10^1^	3.9234 × 10^5^ =	2.4493 × 10^5^	1.5499 × 10^4^ =	1.0559 × 10^4^
DE	1.1568 × 10^3^ +	2.4300 × 10^1^	1.6569 × 10^6^ +	7.9478 × 10^5^	3.0594 × 10^4^ +	2.0254 × 10^4^
GWO	1.8756 × 10^3^ +	7.1996 × 10^2^	6.9142 × 10^7^ +	8.6664 × 10^7^	2.2559 × 10^7^ +	5.8838 × 10^7^
MFO	5.7541 × 10^3^ +	5.0815 × 10^3^	3.7185 × 10^8^ +	5.0196 × 10^8^	9.3223 × 10^7^ +	3.0688 × 10^8^
SCA	2.0663 × 10^3^ +	2.1876 × 10^2^	1.1999 × 10^9^ +	3.7840 × 10^8^	3.7641 × 10^8^ +	1.7320 × 10^8^
PSO	1.2075 × 10^3^ +	3.2471 × 10^1^	3.3489 × 10^4^ −	2.0523 × 10^4^	**1.2396 × 10^4^ =**	1.5268 × 10^4^
PO	1.3055 × 10^3^ +	6.5945 × 10^1^	3.3512 × 10^7^ +	3.5698 × 10^7^	1.4785 × 10^5^ +	1.1272 × 10^5^
BA	1.3242 × 10^3^ +	8.0755 × 10^1^	2.2536 × 10^6^ +	1.7036 × 10^6^	3.0314 × 10^5^ +	1.1221 × 10^5^
FA	3.5574 × 10^3^ +	4.3103 × 10^2^	1.4741 × 10^9^ +	3.8706 × 10^8^	6.0224 × 10^8^ +	1.6634 × 10^8^
	**F13**		**F14**		**F15**	
	**Avg**	**Std**	**Avg**	**Std**	**Avg**	**Std**
CCESC	2.4961 × 10^4^	1.9010 × 10^4^	**5.2832 × 10^3^**	5.9862 × 10^3^	**1.9362 × 10^3^**	1.8960 × 10^2^
ESC	**1.4643 × 10^4^ −**	1.3725 × 10^4^	7.2748 × 10^3^ =	7.5329 × 10^3^	2.0617 × 10^3^ +	2.1360 × 10^2^
DE	6.4083 × 10^4^ +	4.3831 × 10^4^	6.7605 × 10^3^ +	3.7226 × 10^3^	2.0987 × 10^3^ +	1.3508 × 10^2^
GWO	2.1191 × 10^5^ +	3.0916 × 10^5^	3.4786 × 10^5^ +	7.0974 × 10^5^	2.3503 × 10^3^ +	2.6201 × 10^2^
MFO	1.6461 × 10^5^ +	3.2360 × 10^5^	3.0154 × 10^7^ +	1.6484 × 10^8^	3.2261 × 10^3^ +	3.8883 × 10^2^
SCA	1.2213 × 10^5^ +	6.0385 × 10^4^	1.3110 × 10^7^ +	1.1163 × 10^7^	3.5654 × 10^3^ +	2.4475 × 10^2^
PSO	6.2203 × 10^3^ −	3.9012 × 10^3^	7.7850 × 10^3^ +	6.5680 × 10^3^	2.9417 × 10^3^ +	3.9223 × 10^2^
PO	4.2016 × 10^4^ +	2.9247 × 10^4^	3.8824 × 10^4^ +	2.5295 × 10^4^	3.0436 × 10^3^ +	3.1588 × 10^2^
BA	6.5404 × 10^3^ −	4.0829 × 10^3^	9.1369 × 10^4^ +	4.9670 × 10^4^	3.2934 × 10^3^ +	4.4017 × 10^2^
FA	1.9705 × 10^5^ +	8.7921 × 10^4^	6.6901 × 10^7^ +	3.0210 × 10^7^	3.4141 × 10^3^ +	1.5962 × 10^2^
	**F16**		**F17**		**F18**	
	**Avg**	**Std**	**Avg**	**Std**	**Avg**	**Std**
CCESC	**1.7798 × 10^3^**	5.8233 × 10^1^	1.9710 × 10^5^	1.7757 × 10^5^	8.0167 × 10^3^	8.9592 × 10^3^
ESC	1.8868 × 10^3^ +	1.2433 × 10^2^	2.8857 × 10^5^ =	3.3036 × 10^5^	**7.3902 × 10^3^ =**	5.8539 × 10^3^
DE	1.8644 × 10^3^ +	5.9255 × 10^1^	3.2283 × 10^5^ +	1.6230 × 10^5^	7.2213 × 10^3^ =	4.1013 × 10^3^
GWO	1.9867 × 10^3^ +	1.4623 × 10^2^	4.5734 × 10^5^ +	4.0013 × 10^5^	4.0608 × 10^5^ +	4.8087 × 10^5^
MFO	2.5587 × 10^3^ +	2.0382 × 10^2^	3.1724 × 10^6^ +	6.9905 × 10^6^	1.3544 × 10^7^ +	3.6473 × 10^7^
SCA	2.3984 × 10^3^ +	1.2507 × 10^2^	3.0253 × 10^6^ +	1.4421 × 10^6^	2.6512 × 10^7^ +	1.5133 × 10^7^
PSO	2.4593 × 10^3^ +	2.9558 × 10^2^	**1.4883 × 10^5^ =**	8.6525 × 10^4^	1.0108 × 10^4^ =	1.0643 × 10^4^
PO	2.3168 × 10^3^ +	2.2105 × 10^2^	5.6943 × 10^5^ +	3.8407 × 10^5^	6.4285 × 10^5^ +	6.9384 × 10^5^
BA	2.8405 × 10^3^ +	3.2629 × 10^2^	2.2019 × 10^5^ =	1.3705 × 10^5^	6.4719 × 10^5^ +	2.9608 × 10^5^
FA	2.5289 × 10^3^ +	1.0249 × 10^2^	3.6599 × 10^6^ +	1.6108 × 10^6^	9.3336 × 10^7^ +	4.5101 × 10^7^
	**F19**		**F20**		**F21**	
	**Avg**	**Std**	**Avg**	**Std**	**Avg**	**Std**
CCESC	**2.1155 × 10^3^**	6.9545 × 10^1^	2.3516 × 10^3^	6.5957 × 10^0^	**2.4905 × 10^3^**	7.2399 × 10^2^
ESC	2.2513 × 10^3^ +	1.1968 × 10^2^	**2.3285 × 10^3^ −**	8.4475 × 10^0^	3.0059 × 10^3^ +	1.1838 × 10^3^
DE	2.1315 × 10^3^ =	5.9738 × 10^1^	2.4108 × 10^3^ +	7.5239 × 10^0^	3.7545 × 10^3^ +	1.9345 × 10^3^
GWO	2.3525 × 10^3^ +	1.2895 × 10^2^	2.3855 × 10^3^ +	2.5234 × 10^1^	4.5912 × 10^3^ +	1.3127 × 10^3^
MFO	2.6958 × 10^3^ +	2.0705 × 10^2^	2.5007 × 10^3^ +	3.7422 × 10^1^	6.4010 × 10^3^ +	1.5213 × 10^3^
SCA	2.5807 × 10^3^ +	1.4258 × 10^2^	2.5478 × 10^3^ +	1.9573 × 10^1^	8.8320 × 10^3^ +	1.9163 × 10^3^
PSO	2.6068 × 10^3^ +	1.9247 × 10^2^	2.4698 × 10^3^ +	3.5134 × 10^1^	5.1165 × 10^3^ +	2.2689 × 10^3^
PO	2.4862 × 10^3^ +	1.6903 × 10^2^	2.5023 × 10^3^ +	4.4799 × 10^1^	2.7944 × 10^3^ +	1.0815 × 10^3^
BA	2.9724 × 10^3^ +	2.2133 × 10^2^	2.6438 × 10^3^ +	6.9904 × 10^1^	6.7510 × 10^3^ +	1.7252 × 10^3^
FA	2.5833 × 10^3^ +	1.0234 × 10^2^	2.5417 × 10^3^ +	9.7253 × 10^0^	3.8372 × 10^3^ +	1.2854 × 10^2^
	**F22**		**F23**		**F24**	
	**Avg**	**Std**	**Avg**	**Std**	**Avg**	**Std**
CCESC	2.6986 × 10^3^	9.0945 × 10^0^	2.8647 × 10^3^	8.6917 × 10^0^	**2.8898 × 10^3^**	7.5304 × 10^0^
ESC	**2.6880 × 10^3^ −**	1.1336 × 10^1^	**2.8517 × 10^3^ −**	1.0709 × 10^1^	2.8976 × 10^3^ +	1.5369 × 10^1^
DE	2.7545 × 10^3^ +	8.0664 × 10^0^	2.9596 × 10^3^ +	1.1611 × 10^1^	2.8874 × 10^3^ −	2.5098 × 10^−1^
GWO	2.7491 × 10^3^ +	3.8744 × 10^1^	2.9293 × 10^3^ +	5.6197 × 10^1^	3.0034 × 10^3^ +	6.0756 × 10^1^
MFO	2.8435 × 10^3^ +	3.8511 × 10^1^	2.9993 × 10^3^ +	4.2330 × 10^1^	3.3859 × 10^3^ +	5.9192 × 10^2^
SCA	2.9857 × 10^3^ +	3.3269 × 10^1^	3.1556 × 10^3^ +	3.0030 × 10^1^	3.2041 × 10^3^ +	5.2181 × 10^1^
PSO	3.2924 × 10^3^ +	1.9323 × 10^2^	3.3705 × 10^3^ +	1.0796 × 10^2^	2.8805 × 10^3^ −	6.3784 × 10^0^
PO	2.9715 × 10^3^ +	6.9490 × 10^1^	3.1048 × 10^3^ +	6.8115 × 10^1^	2.9227 × 10^3^ +	2.7850 × 10^1^
BA	3.3136 × 10^3^ +	1.5781 × 10^2^	3.3232 × 10^3^ +	1.2567 × 10^2^	2.9127 × 10^3^ +	2.4890 × 10^1^
FA	2.9154 × 10^3^ +	9.4232 × 10^0^	3.0645 × 10^3^ +	1.1776 × 10^1^	3.5518 × 10^3^ +	1.2014 × 10^2^
	**F25**		**F26**		**F27**	
	**Avg**	**Std**	**Avg**	**Std**	**Avg**	**Std**
CCESC	**4.0531 × 10^3^**	1.1532 × 10^2^	3.2141 × 10^3^	9.3902 × 10^0^	3.1715 × 10^3^	6.4540 × 10^1^
ESC	4.0841 × 10^3^ =	1.8511 × 10^2^	3.2302 × 10^3^ +	1.7731 × 10^1^	3.1789 × 10^3^ =	5.3878 × 10^1^
DE	4.6572 × 10^3^ +	1.2058 × 10^2^	**3.2068 × 10^3^ −**	3.3581 × 10^0^	3.1779 × 10^3^ =	5.7495 × 10^1^
GWO	4.7819 × 10^3^ +	3.5736 × 10^2^	3.2393 × 10^3^ +	1.7523 × 10^1^	3.4680 × 10^3^ +	2.3047 × 10^2^
MFO	5.9247 × 10^3^ +	6.3639 × 10^2^	3.2603 × 10^3^ +	2.9104 × 10^1^	4.6385 × 10^3^ +	1.0065 × 10^3^
SCA	6.9798 × 10^3^ +	3.3407 × 10^2^	3.3936 × 10^3^ +	4.5056 × 10^1^	3.8195 × 10^3^ +	9.7138 × 10^1^
PSO	6.3088 × 10^3^ +	2.3791 × 10^3^	3.3135 × 10^3^ =	2.8523 × 10^2^	**3.1467 × 10^3^ =**	6.2260 × 10^1^
PO	6.2389 × 10^3^ +	1.5609 × 10^3^	3.3153 × 10^3^ +	4.9880 × 10^1^	3.3089 × 10^3^ +	5.5567 × 10^1^
BA	8.8007 × 10^3^ +	2.6331 × 10^3^	3.4038 × 10^3^ +	9.5290 × 10^1^	3.1534 × 10^3^ =	6.4405 × 10^1^
FA	6.4862 × 10^3^ +	1.8155 × 10^2^	3.3339 × 10^3^ +	1.6953 × 10^1^	3.8787 × 10^3^ +	9.0911 × 10^1^
	**F28**		**F29**			
	**Avg**	**Std**	**Avg**	**Std**		
CCESC	**3.4364 × 10^3^**	5.4729 × 10^1^	7.3284 × 10^3^	1.8753 × 10^3^		
ESC	3.5012 × 10^3^ +	1.2967 × 10^2^	1.0768 × 10^4^ +	2.2758 × 10^3^		
DE	3.5250 × 10^3^ +	6.9103 × 10^1^	1.1751 × 10^4^ +	3.3232 × 10^3^		
GWO	3.7476 × 10^3^ +	1.7946 × 10^2^	5.8495 × 10^6^ +	5.5302 × 10^6^		
MFO	4.1130 × 10^3^ +	2.4497 × 10^2^	7.5618 × 10^5^ +	1.0771 × 10^6^		
SCA	4.5913 × 10^3^ +	2.9433 × 10^2^	7.0828 × 10^7^ +	3.0432 × 10^7^		
PSO	3.9840 × 10^3^ +	3.2357 × 10^2^	**5.0228 × 10^3^ −**	1.7910 × 10^3^		
PO	4.3708 × 10^3^ +	3.4611 × 10^2^	6.2783 × 10^6^ +	4.9233 × 10^6^		
BA	4.9522 × 10^3^ +	5.0689 × 10^2^	1.2934 × 10^6^ +	7.2635 × 10^5^		
FA	4.7103 × 10^3^ +	1.5665 × 10^2^	9.7735 × 10^7^ +	2.3550 × 10^7^		
	**Overall Rank**					
	**RANK**	**+/=/−**	**AVG**			
CCESC	1	~	2.1034			
ESC	2	11/9/9	2.4828			
DE	3	21/4/4	3.3448			
GWO	5	29/0/0	5.2069			
MFO	8	29/0/0	7.4483			
SCA	9	29/0/0	8.3448			
PSO	4	17/6/6	4.2414			
PO	6	29/0/0	6.1034			
BA	7	24/3/2	7.1379			
FA	10	29/0/0	8.5862			

**Table 3:** The results of CCESC and other algorithms on the oil reservoir production optimization.

Algorithm	NPV (USD)			
	Mean	Std	Best	Worst
CCESC	9.457 × 10^8^	1.532 × 10^7^	9.782 × 10^8^	9.146 × 10^8^
ESC	8.859 × 10^8^	2.510 × 10^7^	9.403 × 10^8^	8.308 × 10^8^
DE	8.986 × 10^8^	3.121 × 10^7^	9.553 × 10^8^	8.391 × 10^8^
GWO	9.053 × 10^8^	2.784 × 10^7^	9.648 × 10^8^	8.557 × 10^8^
MFO	8.247 × 10^8^	4.542 × 10^7^	8.905 × 10^8^	7.692 × 10^8^
SCA	8.704 × 10^8^	3.487 × 10^7^	9.251 × 10^8^	8.107 × 10^8^
PSO	8.552 × 10^8^	3.939 × 10^7^	9.106 × 10^8^	7.895 × 10^8^

## Data Availability

The numerical and experimental data used to support the findings of this study are included with-in the article.

## References

[1] Lin C., Wang P., Heidari A.A., Zhao X., Chen H. (2023). A Boosted Communicational Salp Swarm Algorithm: Performance Optimization and Comprehensive Analysis. J. Bionic Eng..

[2] Cheng M.-M., Zhang J., Wang D.-G., Tan W., Yang J. (2023). A Localization Algorithm Based on Improved Water Flow Optimizer and Max-Similarity Path for 3-D Heterogeneous Wireless Sensor Networks. IEEE Sens. J..

[3] Garg T., Kaur G., Rana P.S., Cheng X. (2024). Enhancing Road Traffic Prediction Using Data Preprocessing Optimization. J. Circuits Syst. Comput..

[4] Kumar A., Das S., Mallipeddi R. (2024). An Efficient Differential Grouping Algorithm for Large-Scale Global Optimization. IEEE Trans. Evol. Comput..

[5] Shan W., Hu H., Cai Z., Chen H., Liu H., Wang M., Teng Y. (2022). Multi-Strategies Boosted Mutative Crow Search Algorithm for Global Tasks: Cases of Continuous and Discrete Optimization. J. Bionic Eng..

[6] Li X., Khishe M., Qian L. (2024). Evolving Deep Gated Recurrent Unit Using Improved Marine Predator Algorithm for Profit Prediction Based on Financial Accounting Information System. Complex Intell. Syst..

[7] Chakraborty A., Ray S. (2024). Economic and Environmental Factors Based Multi-Objective Approach for Optimizing Energy Management in a Microgrid. Renew. Energy.

[8] Hu H., Wang J., Huang X., Ablameyko S.V. An Integrated Online-Offline Hybrid Particle Swarm Optimization Framework for Medium Scale Expensive Problems. Proceedings of the 2024 6th International Conference on Data-driven Optimization of Complex Systems (DOCS).

[9] Liang J., Lin H., Yue C., Ban X., Yu K. (2024). Evolutionary Constrained Multi-Objective Optimization: A Review. Vicinagearth.

[10] Biegler L.T., Grossmann I.E. (2004). Retrospective on Optimization. Comput. Chem. Eng..

[11] Meza J.C. (2010). Steepest Descent. WIREs Comput. Stat..

[12] Polyak B.T. (1969). The Conjugate Gradient Method in Extremal Problems. USSR Comput. Math. Math. Phys..

[13] Hu H., Shan W., Tang Y., Heidari A.A., Chen H., Liu H., Wang M., Escorcia-Gutierrez J., Mansour R.F., Chen J. (2022). Horizontal and Vertical Crossover of Sine Cosine Algorithm with Quick Moves for Optimization and Feature Selection. J. Comput. Des. Eng..

[14] Dantzig G.B. (2002). Linear Programming. Oper. Res..

[15] Bellman R. (1966). Dynamic Programming. Science.

[16] Hu H., Shan W., Chen J., Xing L., Heidari A.A., Chen H., He X., Wang M. (2023). Dynamic Individual Selection and Crossover Boosted Forensic-Based Investigation Algorithm for Global Optimization and Feature Selection. J. Bionic Eng..

[17] Bottou L., Curtis F.E., Nocedal J. (2018). Optimization Methods for Large-Scale Machine Learning. SIAM Rev..

[18] Huang X., Hu H., Wang J., Yuan B., Dai C., Ablameyk S.V. Dynamic Strongly Convex Sparse Operator with Learning Mechanism for Sparse Large-Scale Multi-Objective Optimization. Proceedings of the 2024 6th International Conference on Data-driven Optimization of Complex Systems (DOCS).

[19] Dokeroglu T., Sevinc E., Kucukyilmaz T., Cosar A. (2019). A Survey on New Generation Metaheuristic Algorithms. Comput. Ind. Eng..

[20] Abdel-Basset M., Abdel-Fatah L., Sangaiah A.K. (2018). Metaheuristic Algorithms: A Comprehensive Review. Computational Intelligence for Multimedia Big Data on the Cloud with Engineering Applications.

[21] Katoch S., Chauhan S.S., Kumar V. (2021). A Review on Genetic Algorithm: Past, Present, and Future. Multimed. Tools Appl..

[22] Askarzadeh A. (2016). A Novel Metaheuristic Method for Solving Constrained Engineering Optimization Problems: Crow Search Algorithm. Comput. Struct..

[23] Rajpurohit J., Sharma T.K., Abraham A., Vaishali (2017). Glossary of Metaheuristic Algorithms. Int. J. Comput. Inf. Syst. Ind. Manag. Appl..

[24] Price K., Storn R.M., Lampinen J.A. (2006). Differential Evolution: A Practical Approach to Global Optimization.

[25] Bertsimas D., Tsitsiklis J. (1993). Simulated Annealing. Stat. Sci..

[26] Rashedi E., Nezamabadi-Pour H., Saryazdi S. (2009). GSA: A Gravitational Search Algorithm. Inf. Sci..

[27] Poli R., Kennedy J., Blackwell T. (2007). Particle Swarm Optimization. Swarm Intell..

[28] Dorigo M., Birattari M., Stutzle T. (2006). Ant Colony Optimization. IEEE Comput. Intell. Mag..

[29] Karaboga D., Basturk B., Melin P., Castillo O., Aguilar L.T., Kacprzyk J., Pedrycz W. (2007). Artificial Bee Colony (ABC) Optimization Algorithm for Solving Constrained Optimization Problems. Foundations of Fuzzy Logic and Soft Computing.

[30] Wolpert D.H., Macready W.G. (1997). No Free Lunch Theorems for Optimization. IEEE Trans. Evol. Comput..

[31] Ho Y.C., Pepyne D.L. (2002). Simple Explanation of the No-Free-Lunch Theorem and Its Implications. J. Optim. Theory Appl..

[32] Desbordes J.K., Zhang K., Xue X., Ma X., Luo Q., Huang Z., Hai S., Jun Y. (2022). Dynamic Production Optimization Based on Transfer Learning Algorithms. J. Petrol. Sci. Eng..

[33] Suwartadi E., Krogstad S., Foss B. (2015). Adjoint-Based Surrogate Optimization of Oil Reservoir Water Flooding. Optim. Eng..

[34] Rasouli H., Rashidi F., Karimi B., Khamehchi E. (2015). A Surrogate Integrated Production Modeling Approach to Long-Term Gas-Lift Allocation Optimization. Chem. Eng. Commun..

[35] Yan M., Huang C., Bienstman P., Tino P., Lin W., Sun J. (2024). Emerging Opportunities and Challenges for the Future of Reservoir Computing. Nat. Commun..

[36] Verma S., Prasad A.D., Verma M.K. (2024). Optimal Operation of the Multi-Reservoir System: A Comparative Study of Robust Metaheuristic Algorithms. Int. J. Hydrol. Sci. Technol..

[37] Wang L., Yao Y., Zhao G., Adenutsi C.D., Wang W., Lai F. (2022). A Hybrid Surrogate-Assisted Integrated Optimization of Horizontal Well Spacing and Hydraulic Fracture Stage Placement in Naturally Fractured Shale Gas Reservoir. J. Petrol. Sci. Eng..

[38] An Z., Zhou K., Hou J., Wu D., Pan Y. (2022). Accelerating Reservoir Production Optimization by Combining Reservoir Engineering Method with Particle Swarm Optimization Algorithm. J. Pet. Sci. Eng..

[39] Gu J., Liu W., Zhang K., Zhai L., Zhang Y., Chen F. (2021). Reservoir Production Optimization Based on Surrograte Model and Differential Evolution Algorithm. J. Pet. Sci. Eng..

[40] Chen G., Zhang K., Zhang L., Xue X., Ji D., Yao C., Yao J., Yang Y. (2020). Global and Local Surrogate-Model-Assisted Differential Evolution for Waterflooding Production Optimization. SPE J..

[41] Zhao Z., Luo S. (2024). A Crisscross-Strategy-Boosted Water Flow Optimizer for Global Optimization and Oil Reservoir Production. Biomimetics.

[42] Gao Y., Cheng L. (2025). A Multi-Swarm Greedy Selection Enhanced Fruit Fly Optimization Algorithm for Global Optimization in Oil and Gas Production. PLoS ONE.

[43] Yue T., Li T. (2025). Crisscross Moss Growth Optimization: An Enhanced Bio-Inspired Algorithm for Global Production and Optimization. Biomimetics.

[44] Ouyang K., Fu S., Chen Y., Cai Q., Heidari A.A., Chen H. (2024). Escape: An Optimization Method Based on Crowd Evacuation Behaviors. Artif. Intell. Rev..

[45] Mirjalili S., Mirjalili S.M., Lewis A. (2014). Grey Wolf Optimizer. Adv. Eng. Softw..

[46] Mirjalili S. (2015). Moth-Flame Optimization Algorithm: A Novel Nature-Inspired Heuristic Paradigm. Knowl.-Based Syst..

[47] Mirjalili S. (2016). SCA: A Sine Cosine Algorithm for Solving Optimization Problems. Knowl.-Based Syst..

[48] Lian J., Hui G., Ma L., Zhu T., Wu X., Heidari A.A., Chen Y., Chen H. (2024). Parrot Optimizer: Algorithm and Applications to Medical Problems. Comput. Biol. Med..

[49] Yang X.-S., Gandomi A.H. (2012). Bat Algorithm: A Novel Approach for Global Engineering Optimization. Eng. Comput..

[50] Yang X.-S. (2010). Firefly Algorithm, Stochastic Test Functions and Design Optimisation. Int. J. Bio-Inspired Comput..

